# Cancer and the Environment projects with four First Nations organizations: working together to address concerns about carcinogens in the environment

**DOI:** 10.17269/s41997-021-00571-y

**Published:** 2021-10-20

**Authors:** Alison L. Palmer, Katy Wong-Francq, Eleanor Setton

**Affiliations:** 1 CAREX Canada, Faculty of Health Sciences, Simon Fraser University, Harbour Centre Campus, Suite 2602, 515 West Hastings St, Vancouver, BC V6B 5K3 Canada; 2 Office of the Vice President, Research and International, University of Waterloo, Waterloo, ON Canada; 3 Department of Geography, University of Victoria, Victoria, BC Canada

**Keywords:** Indigenous health, Environmental health, Environmental exposures, Carcinogen exposures, Knowledge to action, Santé autochtone, santé environnementale, exposition environnementale, exposition aux cancérogènes, des connaissances à la pratique

## Abstract

**Setting:**

For First Nations people, human health and well-being are interconnected with a healthy environment. First Nations organizations commonly raise concerns regarding carcinogens in the environment; however, few case studies are available as guidance for working in a participatory and respectful way to help assess and address these concerns.

**Intervention:**

Through four community-led pilot projects executed over two years, we collaborated with 15 participants from four First Nations organizations across four provinces to identify concerns related to environmental carcinogens and to address those concerns through an integrated knowledge translation (KT) approach. We co-developed and implemented strategic KT plans for each pilot project, and conducted evaluation surveys and interviews with participants at multiple time points to assess process, progress, barriers and facilitators, and impact.

**Outcomes:**

The activities and outputs of the pilot projects are available at www.carexcanada.ca. Participants identified 18 concerns, and we co-developed 24 knowledge products. Tailored fact sheets for communities and briefing notes for leadership were deemed most useful; interactive maps were deemed less useful. Evaluation indicated that the collaborative projects were effective in addressing the concerns raised regarding exposures to carcinogens.

**Implications:**

The participant-led approach and multi-year funding to support capacity enhancement and face-to-face engagement were facilitators to project success. However, participants did face important barriers to collaborate which should be considered in future projects of this kind: the most important being a lack of resources (people and time), given competing and often more urgent priorities.

## Résumé

**Lieu:**

Pour les Premiers Peuples, la santé et le bien-être humains sont indissociables de la santé de l’environnement. Les organismes des Premières Nations se disent souvent préoccupés par les cancérogènes présents dans l’environnement, mais peu d’études de cas sont disponibles pour apprendre à travailler de façon participative et respectueuse à évaluer ces préoccupations et à y répondre.

**Intervention:**

Dans le cadre de quatre projets pilotes de proximité menés sur une période de deux ans, nous avons collaboré avec 15 participants, issus de quatre organismes des Premières Nations dans quatre provinces, à cerner leurs préoccupations liées aux cancérogènes dans l’environnement et à y répondre selon une démarche intégrée d’application des connaissances. Nous avons conjointement élaboré et mis en œuvre des plans stratégiques d’application des connaissances pour chaque projet pilote et mené des sondages d’évaluation et des entretiens avec les participants à plusieurs reprises pour évaluer le processus, les progrès accomplis, les éléments favorables et défavorables et les impacts des projets.

**Résultats:**

Les activités et les extrants des projets pilotes sont présentés sur le site www.carexcanada.ca. Les participants ont exprimé 18 motifs de préoccupation, et nous avons élaboré avec eux 24 produits du savoir. Les fiches d’information adaptées à chaque communauté et les notes d’information pour les dirigeants ont été jugées très utiles, mais les cartes interactives un peu moins. Selon l’évaluation, les projets collaboratifs ont réussi à répondre aux préoccupations soulevées quant à l’exposition aux cancérogènes.

**Conséquences:**

La démarche axée sur les participants et le financement pluriannuel consacré au renforcement des capacités et aux contacts directs ont été des éléments favorables à la réussite des projets. Par contre, les participants ont fait face à d’importants obstacles à la collaboration dont il faudrait tenir compte dans les futurs projets de la sorte, le principal obstacle étant le manque de ressources (personnes et temps), étant donné l’existence de priorités concurrentes et souvent plus urgentes.

## Introduction

For First Nations people, human health and well-being are interconnected with the health of the land, air, water, plants, and animals. Discussions about Indigenous health often involve concerns about environmental quality and contaminants in the food and wildlife harvested locally (Assembly of First Nations, 2008; Sharp et al., 2016). Several contaminants programs and research studies have investigated these concerns (Chan et al., 2019; First Nations Health Authority, 2020; Government of Canada, 2020a, b). As a result, some community-specific and local data on contaminants exist; however, evidence linking environmental contaminants and health outcomes, such as cancer, among First Nations in Canada is limited (Mazereeuw et al., 2018).

Updated national information on the incidence of cancer and mortality in First Nations is also lacking; much of the available data are over 10 years old, making it difficult to understand current cancer realities for First Nations people (Canadian Partnership Against Cancer, 2013). Life expectancies for First Nations men and women in Canada are lower, respectively, than those for non-First Nations men and women (Tjepkema et al., 2009). Regional studies show that First Nations people have lower survival rates for many of the most common cancers (McGahan et al., 2017; Withrow et al., 2017) and disproportionately higher rates of certain cancers, including colorectal, kidney, cervical, and liver cancers, as well as lung cancer in some geographic regions (Mazereeuw et al., 2018; McGahan et al., 2017). The future cancer burden is expected to be high in the First Nations on-reserve population in particular (Elias et al., 2011). These realities are in contrast to the past, when First Nations people in Canada had lower cancer incidence and mortality rates than non-First Nation populations (Elias et al., 2011). While these increases can be explained partly by longer life expectancy, as for all populations, and a large First Nations youth population that will increase the cancer burden as they age, an interplay of genetic, social, and environmental factors influences cancer rates as well.

When increased cancer rates are observed within a community or when a cancer cluster is suspected (a larger-than-expected number of cancer cases occurring within a group of people in a geographic area over a period of time), attention often turns to environmental pollution as a potential cause (Goodman et al., 2012). When such a concern is identified by a community, the burden of proof is often placed on that community to demonstrate the risk to its health, and then push for action. Similarly, when a resource development project is proposed near or through its territory, a First Nations community must engage in the environmental or health impact assessment processes to raise its concerns about potential impacts. The challenges around these activities are well documented (Assembly of First Nations, 2008).

CAREX Canada (http://www.carexcanada.ca) and the former Spatial Sciences Research Lab (SSRL) at the University of Victoria collaborated with the Assembly of First Nations (AFN) Environment Unit and the First Nations Environmental Health Innovation Network (FNEHIN) from 2009 to 2016 to enhance First Nations’ capacity in environmental health, specifically regarding carcinogen exposures. This work sought to increase opportunities for First Nations organizations to access complex datasets and identify priorities for action on a substance-by-substance and regional basis (Chan et al., 2019; Setton et al., 2015).

The Cancer and the Environment (C&E) projects focused on working with First Nations organizations to identify concerns regarding carcinogens in the environment and to pursue knowledge translation (KT) activities to help address those concerns. A secondary goal was to gain insights into useful approaches for supporting First Nations’ enhanced understanding of carcinogen exposures and enhanced capacity for action. KT and community-based participatory research approaches form the foundation of the C&E projects described within (Bharadwaj, 2014; Cargo & Mercer, 2008; Graham et al., 2006; Lemire et al., 2013).

## Methods

In 2013, CAREX and SSRL used a Meetings and Dissemination grant from the Canadian Institutes of Health Research (CIHR) to convene a First Nations KT Advisory Committee. The committee developed a strategic framework and plan for building on previous work and helping to address concerns about environmental health in First Nations communities (First Nations Knowledge Translation Advisory Committee, 2013). The strategic plan was guided by the following principles: use results responsibly, support self-determination, stay connected to spirituality, enhance capacity, and be ethical (follow OCAP® principles of ownership, control, access, and possession) (First Nations Information Governance Centre (FNIGC), 2020). CAREX and SSRL applied this strategy with the C&E projects with the support of a 2-year CIHR Knowledge-to-Action grant that engaged the Propel Centre for Population Impact (now closed), University of Waterloo, as a collaborator in addition to the AFN.

To begin the projects, the research team and the AFN released a call for C&E projects to over 650 First Nations organizations and communities across Canada. Out of 16 C&E proposals received, the First Nations Knowledge Translation Advisory Committee chose five projects from around the country (First Nations Knowledge Translation Advisory Committee, 2013), of which one had to withdraw early.

Moving forward with four projects with four organizations (two communities and two organizations representing or working with various communities) across Alberta, Manitoba, Ontario, and Quebec, each organization appointed or nominated two to five members to their project team, to collaborate with the research team. These members held various roles related to health, environment, and land management. Our policy analyst partner from the AFN ensured that First Nations protocols were followed throughout all project activities, including events.

The project’s approach centred on the following guiding principles:

- **Collaborative and participatory:** engage all participants throughout project design and implementation (Cargo & Mercer, 2008).
- **Culturally appropriate:** design projects with First Nations participants to ensure culturally appropriate strategies and activities, and to support self-determination (Banister et al., 2011; Government of Canada, 2018).
- **Utilization-based:** evaluate projects with a focus on assessing utilization for learning and impact (Patton, 2008).
- **Methodologically appropriate and of high quality:** collect, manage, and interpret data in a manner best suited to answer the evaluation questions, and adhering to OCAP® principles.
- **Ethically sound: **have data collection procedures reviewed by the Ethics Review Board at the University of Victoria and University of Waterloo.

The first event was an introductory webinar for all team members to clarify guiding principles (including ethics), overall goals, and timeline; convey the types of expertise offered by the research team; and present the resources and tools available from CAREX Canada.

Following this, a face-to-face strategic planning workshop was organized in Winnipeg, Manitoba, attended by all project and research team members. This event demonstrated the CAREX Canada offerings on exposures to carcinogens in more detail and developed draft strategic KT plans tailored to the exposures of concern identified by each project. Priorities and concerns were openly raised by participants; participants were not led with categories or drop-down lists to narrow the scope to carcinogens only given the mandate of the CAREX Canada project. A paper survey was distributed to participants to anonymously evaluate the event presentations, objectives, design, and results using closed- and open-ended questions. Responses were used to inform the development of future events.

As work commenced to deliver the strategic plans, a common need was identified for another event—a more thorough introduction to cancer to address gaps in understanding on causes, risk factors, and prevention guidance. This workshop took place in Toronto, and was offered with Ontario Health’s Indigenous Cancer Care Unit, which has since developed a “Cancer 101 Toolkit” with the materials (Ontario Health, 2020). Participants were surveyed anonymously on the event to inform future events.

Semi-structured evaluation interviews were conducted by phone at the project mid-point with eight project participants. The goal of these interviews was that participants:

1. Assess their progress towards meeting the project objectives (as per the strategic plan determined for each project in the initial workshop) and identify barriers and facilitators to meeting them;
2. Obtain feedback on process, including whether projects were participatory, respectful, and paced appropriately; and
3. Assess outcomes to date, anticipated outcomes, and future intentions for project results.

Interviews were transcribed and results were analyzed using thematic content analysis. The themes identified were strongly linked to the questions and responses. As a result, an inductive approach to data analysis was used whereby high-level conclusions were drawn instead of fitting the data into a pre-existing theory or framework.

The KT activities outlined in the strategic plans involved knowledge synthesis (with C&E project teams providing local background and context to the exposure of concern, and the research team developing corresponding knowledge products, such as briefing notes, fact sheets, brochures, and reports) and GIS analysis (to develop interactive spatial data browsers). As activities were underway, we offered one in-person workshop with each project group to present sample knowledge products; assess gaps in those products as well as language accessibility and format usability; and solicit input on additional products the group might find useful in the remaining timeframe. This event was also surveyed, and responses analyzed.

Once all strategic plans were executed and resources co-developed, we conducted semi-structured, end-of-project evaluation interviews by phone with 10 participants. The goal was to have participants:

1. Assess project benefits and challenges;
2. Assess whether the content of the resources developed met organization/community needs (in their opinion and where appropriate, based on discussions with community leadership and community members);
3. Rank and comment on the activities and resources in terms of usefulness, and identify which resource(s) was most/least useful or had the most/least impact, and why (for least useful, identify what could have been done differently);
4. Assess impacts of project involvement (e.g., increased community awareness, enhanced capacity, influence on decision-making, practice, and policy at organization or community level, application to training programs).

Interviews were transcribed and results were analyzed using thematic content analysis (Bernard & Ryan, 2009), as described above.

## Results

### Strategic KT products

The objectives and outputs of the strategic KT plans for each project are summarized in Table 1. These varied depending on the objective, needs, and priorities of the organization. The 24 KT products relate to 18 concerns identified by participants on behalf of the communities and community members they work with. Most related to exposure sources and pathways. Common concerns included exposures associated with existing and potential industrial emitters, contaminants in traditional foods, and radon gas exposure in homes. Briefing notes and reports were the most-requested product.

**Table 1:** List of resources co-developed through the Cancer and Environment projects (available on the CAREX website: http://www.carexcanada.ca)

Project objectives	Output / KT product	Description
PROJECT 1: The focus of this project was synthesizing knowledge to help lands and resource staff better understand environmental health issues in the region	Interactive Map (Data browser)	An online map showing the major emitters, active and inactive mine sites, federal contaminated sites, major rivers, and watersheds in the region. A manual for using this map was also developed
	An overview of contaminant sources and measurements, stakeholders, and potential future emissions in the region (Report)	Summarizes data from publicly available sources on the number of large emitters and contaminated sites within 50 km of each First Nations community
	Environmental pollutants - Sources (Report)	Shows how close emitters and contaminated sites are to First Nations communities
	Environmental pollutants - Monitoring (Report)	Investigates whether measured levels are of concern for increased cancer risk
	Regional stakeholders in resource development or protection of human health (Report)	Lists contact information for a wide range of relevant organizations in the region
	Future regional resource development (Report)	Conveys which known and suspected carcinogens are emitted by different sectors, and information about pending and recently permitted projects with potential to increase emissions
PROJECT 2: The focus of this project was developing community outreach materials and enhancing capacity to look at specific concerns such as drinking water quality and radiation exposure	Interactive Map (Data browser)	An online map showing the major emitters, active and inactive mine sites, federal contaminated sites, major rivers, and watersheds in and around the First Nation territory. A manual for using this map was also developed
	Cancer education activity (toolkit)	An education kit aimed at children ages 8–14 to increase awareness of risk factors for cancer, including a series of cards covering five different cancer risk factors: smoking, activity level, diet, pollution, and screening
	Drinking water chlorination - What are the risks? (Briefing note)	Discusses the balance between the use of chlorine-based additives to kill harmful organisms in drinking water and the levels of harmful chemicals created as by-products in the disinfection process
	Polycyclic aromatic hydrocarbons in traditional foods (Briefing note)	Looks at the cancer risk associated with PAHs in the most commonly consumed traditional foods
	Radon (Briefing note)	Reviews why radon levels are naturally higher in some areas of Canada, how exposure occurs, and what can be done about it
PROJECT 3: The focus of this project was increasing community awareness and enhancing capacity to look at specific concerns such as radon exposure in homes and contaminants in traditional foods	Interactive Map (Data browser)	An online map showing the major emitters, active and inactive mine sites, federal contaminated sites, major rivers, and watersheds in and around the First Nation territory. A manual for using this map was also developed
	Traditional food - Berries (Fact sheet)	Describes the health benefits of eating berries, as well as the precautions to take when picking them
	Traditional food - Meat (Fact sheet)	Looks at the difference between caught and bought meat, and interprets data on levels of heavy metals
	Burning wood and garbage: Why not? (Fact sheet)	Outlines the many harmful gases and chemicals emitted through burning wood and garbage
	Pesticides - Glyphosate (Briefing note)	Summarizes the latest research on health effects of glyphosate, currently the most highly produced herbicide in the world
	Development projects - Hog farms (Briefing note)	Investigates whether any cancer-causing substances are produced by large scale hog farming
	Development projects - Mining rare earth elements (Briefing note)	Outlines some of the possible health and environmental impacts of a rare earth element mine
	Radon - Monitoring in homes (Brochure)	Answers the questions that community members may have about radon sampling in their homes
	Radon - Interpreting test results (Brochure)	Provides a useful guide for interpreting home test results for radon
PROJECT 4: The focus of this project was increasing member communities’ awareness and enhancing capacity to look at specific concerns such as cancer cluster investigations and firefighters' exposures	Pollution - Is it causing cancer in my community? (Briefing note)	Presents some of the key questions asked during a cancer cluster investigation, which can be used as a guideline for better understanding cancer rates in a community
	Firefighters - Three reasons why using your self-contained breathing apparatus (SCBA) makes sense (Briefing note)	Outlines the many toxic substances found in smoke, why firefighters have higher rates of some cancers, and how an SCBA reduces exposure
	Metformin - Are there impacts to my health and the environment? (Briefing note)	Looks at the potential impacts of metformin, one of the most frequently prescribed medications for treating type 2 diabetes

All the resources are available at https://www.carexcanada.ca/special-topics/first-nations/. We also compiled and shared on the CAREX website resources developed by First Nations and other organizations on topics ranging from cancer screening and treatment to traditional food and tobacco.

### Evaluation — mid-point and final interviews

The high-level mid-point interview results are summarized in Table 2 and final interview results are summarized in Table 3.

**Table 2:** Results from mid-point evaluation interviews with Cancer and the Environment project participants

Theme	Subtheme	Response
Progress towards objectives	Status of project objectives	Some progress had been made by the various project groups on their objectives; however, several participants noted that their projects were not moving along as rapidly as anticipated
	Barriers to progress identified	*Time and financial constraints*: being too busy, having too many other competing priorities, having crises to manage, being short on staff, lack of financial resources
		*Internal/resource challenges*: lack of project management/leadership internally, lack of IT support internally, lack of environmental and technical expertise (among participants personally as well as internally among colleagues)
		*Logistics*: geography (team members working at different sites, distance from CAREX team), finding a suitable and available contractor for support, contractor retention and follow-through
		*External challenges*: lack of support from government (existing environmental health officers at federal level having too many communities to support, government funds not being put towards environmental health support at community, Tribal council, or territory organization levels)
	Facilitators to progress identified	*Regular, constructive interactions with research team:* being in contact with CAREX team more often (e.g., having more phone conferences), setting joint deadlines with CAREX staff, co-developing a detailed workplan with built-in contingency plans, having a face-to-face session with CAREX team in summer, having CAREX team support some objectives more directly (e.g., looking for measurement data), having continuous dialogue about what is working and what’s not
		*Internal/resource improvements*: increasing staff capacity to work on objectives, getting together as an internal team more consistently, engaging other internal departments (e.g., lands), putting internal deadlines on objectives
		*Resources:* having a few slides or a one pager on the project (i.e., to pursue opportunities that arise to talk about project with the community), putting information in existing meeting packages for leadership
		*Strategies:* providing a lunch to engage health technicians in a presentation on results from first objective, identifying a few champions of the projects at the regional level, considering seasonality in project delivery (e.g., when berries emerge or hunting begins, more discussions about environment), having materials prepared to share when a death from cancer occurs in the community, considering youth as an audience (e.g., using youth centre)
Feedback on process	Has the process been participatory	All participants found the process participatory. Many noted that that they had been consulted, that interaction was consistent, and that there was good dialogue, particularly at the workshops “I think if we didn’t have you guys on board, we probably would not be doing what we are doing so I have to say ‘yes’.” However (as noted under progress), many felt that more interaction with the research team would be beneficial
	Has the process been respectful	All participants found the process respectful. Many appreciated the attention to prayer and ceremony at the workshops, and the way the principles of OCAP® were considered and applied throughout “Oh yeah, definitely…what I noticed was you had asked: ‘What do you want to do?’ ‘How do you want to do it?’ ‘Where do you want to stay?’ That is respectful in itself to the people who were attending.” “I think it has been a respectful process. I like that you guys have taken a lead and taken control of how this moves.”
	Has the pace been appropriate	Participants found the pace “good” or “just right” as far as process, although a bit slow as far as meeting objectives. “To me, it’s at a good pace. We don’t want to have people push you and push you because then we tend to not get things done, but I think it’s been at a good pace. Technically, I think we would have moved a bit faster if we had been more prepared here.”
Feedback on outcomes	Main outcomes so far	All participants noted that their awareness and knowledge of cancer had increased, along with knowledge about CAREX tools and resources. Some expressed feeling more active on the topic in their work, and more comfortable sharing information. Several participants noted that silos between departments were being broken down at their respective organizations through this work. Learnings from other project groups was another positive outcome shared by several participants “I feel that I would call you if I needed some help…before I didn’t have anyone on speed dial but [now] I do, so that’s a good thing.” “I like learning what other people were doing in other regions and some of the different projects and scopes that were being talked about in our initial sessions. For me that is an invaluable part of the project - the networking with other people in other regions.”
	Anticipated outcomes in future	Most participants anticipated increased education and knowledge about cancer and the environment at the community level. Increased leadership awareness was another anticipated outcome. Various secondary outcomes were anticipated, including more communities informed and empowered to protect the environment, more people engaged and talking about cancer, and more people being proactive about their health. Many participants expressed a hope that this work would lead to lower cancer rates “It is a really isolated approach right now where communities work in isolation of each other so certain areas might have discussions about hydro and then they will go to another community, so they are really isolated. So, I think opening up this discussion to a broad audience is going to be beneficial as well.”
	Future intentions with regard to project objectives	Various intentions were shared by participants, including communicating the gaps in knowledge identified by the project and encouraging communities to fill those gaps, encouraging dialogue between parents and kids about cancer to support early conversations, and linking up this work with federal government priorities on environmental protection to ensure the project informs or feeds into future work

**Table 3:** Results from final evaluation interviews with Cancer and the Environment project participants

Theme	Responses
Benefits	• Provided important information to community about topics of interest and also about cancer in general (determinants and causes, and how to look for appropriate information) • Briefing notes were used by department and leadership, including health centre and vice-chief; they were useful for negotiations, consultations (for example, consultations were ongoing at the time the briefing note came out, so was timely and helpful) • Learned more about food in the area and potential carcinogens in the environment • Learned more about certain development projects in the area • Having face-to-face workshops provided time away from the office to focus on this topic “I think back to the times that we did actually sit down and make some progress it was because we actually booked the time and we got stuff prepared and we were actually away from the office here too, because when we’re here, we get pulled in 50 different directions and we never can get things done.”
Challenges	• Resources (among participants and audiences): participants spread thin, covering many diverse and complex file areas, competing priorities (especially those that are crisis-related), feeling like an additional staff person is required to focus on the project, hard to engage communities for consultation and uptake given demands and limited staff “Always a challenge is time management, just because we’re spread so very thin. We cover a lot of diverse and complex file areas, so that for me has been the biggest challenge.” • Finding the appropriate avenues to communicate project findings • Communicating the limitations of science in looking at environmental exposures and cancer (addressing the perception in communities that there’s a direct correlation between resource development, resource exploitation, and cancer) • Communicating the science behind the projects to leadership and communities • Distance between participants on each project and with research team
Resources developed	• Participants ranked 91% of project activities and resources “very useful” or “useful”; 9% were ranked as “somewhat useful” • The most useful resources were fact sheets (distributed in community newsletters) and briefing notes (used in consultations, negotiations) • Fact sheets and brochures were simple and to the point “If it’s plain and simple, a fact sheet would be probably be about the best that’ll work in our community and have a little bit more of an impact on our community members.” • Detailed reports were useful for discussions with leadership, strong and simple executive summaries supported staff in presenting those reports • The least useful resource was the interactive map • By working together, we were able to nail reading level (at first the language was too scientific) • Analogies worked well to explain technical topics
Outcomes	• Projects influenced funding applications, future work plans, and comprehensive community planning • Painted an evidence-based picture of potential issues associated with past and proposed resource development projects (which leadership suspected but did not have a fulsome picture of) “It gave them [leadership] the opportunity to reflect on what’s happening in their community. I think they get so busy sometimes. So, us saying, ‘We’ve done this project. We’ve outlined where we’ve had some resource development in the area, where there are some potential sites coming, to prepare those communities.’ I think it started to shake up the conversation, and make people focus on comprehensive community planning, and ensuring that what you have is protected.” • Participants increased awareness of and access to specialized knowledge • Enhanced capacity at participant organizations to talk about cancer and the environment, translate to communities “Yes. We have enhanced our capacity through the workshops with CAREX and received a lot of information that is going to be helpful in the future. The more information we know about the land, the better.” • Increased conversations at the community level about topics such as wood burning, risk factors, screening” • Increased networking with external associations, tribal councils, and communities on the topic
Value added	• “It filled holes and provided information that was out there but that we had no time to seek out.” • “Great opportunity to listen to other groups, learn about what they’re looking at, what their priorities are – networking function.” • “We felt the CAREX information and the project were very worthwhile, and needed.” • “ I like the fact that there is information provided that’s accurate, that it’s not through a government agency, so it’s a little bit easier to sell to people.”
Next steps	• Participants will present to neighbouring communities at a tribal meeting, for example • Participants will present to tribal council health directors or at least share the resources (via the CAREX website and through a health portal in development) • Participants will explore opportunities to share resources with an Elders Committee, land managers association, and Youth Centre

## Discussion

### Strengths and limitations

The evaluation identified the collaborative, partner-led approach as a strength of the projects. The two-year funding was another strength, facilitating face-to-face engagement that supported relationship- and trust-building crucial to meaningful collaboration and producing useful products. Having in-person workshops was critical; they provided participants time away from the office to overcome competing priorities. The funding supported three types of in-person events, two workshops with the full group of project members and the full research team, plus four smaller workshops with each project group individually and two research team members. The smaller workshops were offered at the request of project teams as per the mid-point interviews.

Another strength was the responsive and adaptive nature of the projects. For example, the initial scope of the projects focused on training programs or toolkits on CAREX resources and tools; however, it became clear in the evaluation survey from the first workshop that a broader focus on knowledge synthesis and concerns regarding cancer and the environment was more appropriate and more useful to C&E project teams. More regular check-ins were initiated by the research team after mid-point interviews indicated this was desired by project teams. In addition, a well-received workshop on “What is cancer?” was organized in response to requests for more background information on cancer, including causes, risk factors, and prevention guidance.

The key limitation for our First Nations partners was a lack of resources (people, time). This limitation affected their ability to participate in the projects as well as share and apply the knowledge products developed. Participants identified various reasons for this, including being too busy, having too many other competing priorities, managing crises, being short-staffed, and/or lacking financial resources. Internal challenges were also identified, such as lack of project management/leadership internally, lack of IT support internally, and lack of environmental and technical expertise (among participants personally as well as internally among colleagues). Although the research team addressed some of these challenges, such as by offering project management support, facilitating more face-to-face time to allow partners to leave their offices to focus on the projects, and providing some financial resources, participants’ limited time to collaborate affected the degree to which the resources could be tailored to maximize uptake. It also affected their time available to share and apply the resources. For example, the resources could have better integrated Indigenous ways of knowing and traditional knowledge as well as strengths-based framing (Institute of Health Economics, 2011). Providing more financial support to the project teams could have helped to overcome some resource limitations. However, we experienced and heard from partners that many of these challenges are systemic for Indigenous organizations and communities, particularly when working with specialized professionals in high demand. For example, efforts to engage local contractors to support project teams with specific objectives were not successful; finding suitable contractors was challenging, and when found, it was difficult to retain contractors to ensure follow-through on project tasks.

Geography was another limitation, given that research team members were primarily based in British Columbia, and project team members were based across the country. Despite the in-person workshops, participants felt that more face time would have contributed to stronger relationships, more opportunity for partners to focus on the project (as opposed to their competing priorities), and potentially more successful projects.

### Learnings and future work

Participants ranked 91% of project activities and resources “very useful” or “useful”; 9% were ranked as “somewhat useful.” Evaluation interviews indicated that the most useful resources were fact sheets on key topics, including the safety of traditional foods, impact of burning wood and garbage, and testing for radon gas. These resources were considered simple, to-the-point, and useful for sharing with the community. Briefing notes on timely issues were deemed very useful for discussions at department and leadership levels as well as in negotiations and consultations. The interactive maps were underused across all projects. These maps used GIS to indicate the locations of major emitters, active and inactive mine sites, federal contaminated sites, major rivers, and watersheds in and around specific territories. One barrier to uptake was the information density of the maps; Internet connectivity was another. Better understanding of what kinds of maps would be most useful for First Nations organizations to understand environmental exposures is the subject of future research. Research questions could include the following: could these maps incorporate traditional knowledge or stories to place the data in a more relevant context? Could other local data sources be considered?

Future C&E projects could seek to embed a strengths-based (versus deficit-based) perspective (Hyett et al., 2019; Thiessen et al., 2020) and Two-Eyed seeing approach into the model (Institute of Health Economics, 2011). They could also take a more focused approach to look at common concerns with several groups, to have a broader impact in addressing those concerns in different regions. This proposal arose out of feedback from project participants, who appreciated meeting as a large group to share experiences and priorities, and to develop a network. Out of the 18 different concerns identified by these project participants, the most common were exposures associated with existing and proposed industrial emitters, contaminants in traditional foods, and radon gas exposure in homes. Since these projects were completed, the CAREX Canada team has received several queries about landfill management and potential related exposures. A companion piece to any future C&E projects could contextualize the queries and concerns, and associated knowledge products developed, within epidemiological data regarding cancer incidence and survival rates.

## Conclusion

These two-year Cancer and the Environment projects offer four case studies for collaborating with First Nations organizations in a meaningful way to help assess and address exposures to carcinogens in the environment. Evaluation indicated that the projects were participatory, respectful, and effective. Impacts include participants’ increased awareness of and access to specialized knowledge about cancer and the environment; enhanced capacity at participant organizations to talk about cancer and the environment, and translate that knowledge to communities; increased conversations at the community level about topics such as wood burning, risk factors, and screening; and increased networking with external associations, tribal councils, and communities on the topic. The knowledge translation products are available on the CAREX website (https://www.carexcanada.ca/special-topics/first-nations/) and have been distributed widely through conferences, meetings, social media, and partners to other First Nations organizations across Canada.

## Implications for policy and practice

What are the innovations in this policy or program?

- These pilot projects offer four unique and distinct case studies for working in collaboration with First Nations organizations to help identify and better understand potential exposures to carcinogens in the environment.
- Applying well-established knowledge translation and community-based participatory research models, these projects were the first of their kind in Canada focused on environmental concerns.
- Through a robust evaluation plan, we worked with our First Nations partners to assess process, progress, barriers and facilitators, and impact of each project. The results of this evaluation contribute valuable learnings to those interested in undertaking successful projects of this kind in future.

What are the burning research questions for this innovation?

- The research question at the outset of these projects was as follows: how can we contribute meaningfully to enhancing First Nations’ capacity in environmental health, specifically around exposures to carcinogens? Through our evaluation, we gained valuable insights into this question in terms of process, barriers and facilitators to collaboration, and suggested changes for future iterations.
- A lack of resources (people and time) among participants was a significant barrier to collaboration. To scale up these projects, we need to better understand how to address this barrier in a practical and sustainable way, given system-level barriers such as funding structures and government priorities.

## Data Availability

N/A

## Code availability

N/A
